# Foraging Movement Patterns of Lactating Mexican Long‐Nosed Bats in Central Mexico

**DOI:** 10.1002/ece3.72055

**Published:** 2025-09-02

**Authors:** Paulina Soriano‐Varela, Ana Ibarra‐Macías, Alberto E. Rojas‐Martínez, Claudia Elizabeth Moreno, Iriana Zuria

**Affiliations:** ^1^ Centro de Investigaciones Biológicas Universidad Autónoma del Estado de Hidalgo Pachuca de Soto Hidalgo Mexico; ^2^ Bat Conservation International Austin Texas USA

**Keywords:** foraging behavior, GPS, *Leptonycteris*, movement patterns, nectar‐feeding bats, forrajeo, GPS, *Leptonycteris*, murciélagos nectarívoros, patrones de movimiento

## Abstract

The Mexican long‐nosed bat ( *Leptonycteris nivalis* ) is a nectar‐feeding bat distributed seasonally between Mexico and the United States, and it has been declared an endangered species in both countries. Here, we describe for the first time the movement patterns and locations of foraging areas used by lactating females from the only known maternity roost in central Mexico. GPS loggers were placed on 29 lactating females, adhered to the interscapular area with short‐term surgical glue. We obtained movement tracks of at least one night for 21 different individuals. Movement patterns were identified using the first passage time segmentation method and classified into commutative and foraging flights. Bats made up to three trips on the same night, visiting between one and three foraging areas. On average, the total distance traveled was 61.72 km per night (minimum 23.11 km, maximum 160.55 km), and 37 foraging areas were identified, located between 13 and 40 km north of the roost, mainly in desert shrublands, followed by agricultural areas and temperate forests. In these places, they spent most of their time outside the roost (2.16 h mean ± 1.14 h SD), feeding on the resources available in an average area of 0.38 km^2^. Bats traveled long distances each night, using areas with abundant wild and human‐cultivated floral resources, reflecting the importance of integrating movement ecology for the design of conservation and habitat management strategies that ensure the availability of necessary resources for this species.

## Graphical abstract

We tracked 21 lactating females of a migratory and endangered nectar‐feeding bat using GPS devices, and analyzed their foraging movements in one of the most important maternity roosts for the conservation of the species. Bats foraged within 50 km from the roost, made up to three foraging trips on the same night, and visited multiple foraging grounds with an average area of 0.38 km^2^.

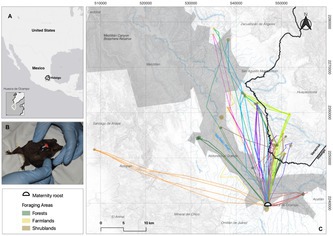

## RESUMEN

*El Murciélago Magueyero Mayor (Leptonycteris nivalis),* es un murciélago nectarívoro que se distribuye estacionalmente entre México y Estados Unidos, la especie está declarada en peligro en ambos países. En este trabajo, describimos por primera vez los patrones de movimiento y las ubicaciones de las áreas de forrajeo de hembras lactantes en el único refugio de maternidad conocido en el centro de México. Se colocaron dispositivos GPS en 29 hembras lactantes, adheridos al área interescapular con pegamento quirúrgico de corta de duración. Se obtuvieron rutas de movimiento de al menos una noche para 21 individuos diferentes. Los patrones de movimiento se idenfiticaron utilizando el método de segmentación pasaje de primer tiempo y se clasificaron como vuelos conmutativos y de forrajeo. Los murciélagos realizaron hasta tres viajes en la misma noche y visitaron entre una a tres áreas de forrajeo. En promedio, la distancia total recorrida fue de 61.72 km por noche (mínimo 23.11 km, máximo 160.55 km). Se identificaron 37 áreas de forrajeo localizadas entre 13 y 40 km hacia el norte del refugio, principalmente en matorrales xerófilos, seguido de áreas agrícolas y bosques templados. En estos lugares, los murciélagos pasaron la mayor parte de su tiempo fuera del refugio (2.16 h mean ± 1.14 h SD), aliméntadose de los recursos disponibles en un área promedio de 0.38 km^2^. Los murciélagos recorren grandes distancias cada noche, utilizando áreas con abundantes recursos florales silvestres y cultivados, reflejando la importancia de integrar la ecología del movimiento en el diseño de estrategias de conservación y manejo de hábitat que aseguren la disponibilidad de los rercusos necesarios para la especie.

## 1 Introduction

The movement of animals throughout the environment and the location of adequate resources for survival are central topics in ecology and conservation biology. Movement is a fundamental characteristic of life, and it affects almost all ecological levels since animals move to gain energy, seek safety, or reproduce (Nathan et al. 2008; Shaw 2020). The above has led to an increase in research on animal movement over time, providing a better understanding of animal ecology and enhancing decisions towards the management and protection of wildlife populations, especially for threatened species, status assessments, and recovery plans (Fraser et al. 2018; Nathan et al. 2008; Pinaud 2008). Through powered flight, bats display a wide capacity for long‐distance movements, with several species from temperate zones migrating seasonally between areas where they give birth to their offspring, hibernate, reproduce, or make nightly movements to reach adequate foraging areas (Fleming 2019; Voigt et al. 2017). Among vertebrates, bat migration is a relatively rare phenomenon, and much less is known about their individual movements during the journey than in other migratory groups such as birds (Fleming 2019; Holland and Wikelski 2009). This is because studying animal movement follows the development of tracking technology, although recent technological advances, such as miniaturized radio transmitters, on‐board loggers, acoustic transmitters, and light‐level geolocators, are gradually providing high‐resolution data, allowing us to understand bat movements at different temporal and spatial scales (Edelhoff et al. 2016; Getz and Saltz 2008; Holland and Wikelski 2009; Hurme et al. 2019; Pinaud 2008; Voigt et al. 2017).

The Mexican‐long nosed bat ( *Leptonycteris nivalis* ) is a nectar‐feeding species that moves seasonally over hundreds of kilometers each year between central Mexico and the southwestern United States, following an agave and cacti nectar corridor (Arita and Santos‐del‐Prado 1999; Fleming 2019; Gómez‐Ruiz and Lacher 2017; Moreno‐Valdez et al. 2004; Valiente‐Banuet et al. 1996; Voigt et al. 2017). The species is listed as federally endangered by the Endangered Species Act of the United States (U.S. Fish and Wildlife Service 1973, 1988), NOM‐059‐SEMARNAT‐2010 in Mexico (Diario Oficial de la Federación 2010), and the IUCN Red List of Threatened Species (Medellín 2015). The main threats for this species survival include human disturbance and the destruction of roosts, loss of food resources due to land use change and harvesting of agave plants, and loss of habitat connectivity that supports annual migratory movements (Medellín 2015; Trejo‐Salazar et al. 2016; U.S. Fish and Wildlife Service 2018). What we know about their movement ecology is still scarce, and migration patterns have been interpreted in terms of how they use the known roosts throughout their distribution (U.S. Fish and Wildlife Service 2018). During winter, adults remain in central Mexico in the only known mating roost along the species distribution, with females leaving by spring and moving north to give birth in maternity roosts (Sánchez and Medellín 2007). So far only five maternity roosts are known along the species distribution, four of which are located between northern Mexico and the southwestern United States, and one is located in central Mexico (U.S. Fish and Wildlife Service 2018). The latter, locally known as “The Tunnel of the Bats,” and hereafter called “Aguacatitla Tunnels,” is considered to be an atypical maternity roost, with bats possibly remaining in central Mexico after the mating season (Rojas‐Martínez et al. 2010; Zamora Vera 2015), because of the great diversity of plant communities from arid zones that provide floral resources throughout the year (CONANP 2003; Rojas‐Martínez et al. 1999, 2021).

Nightly movements and local activity patterns of Mexican long‐nosed bats have been studied at Mount Emory Cave and Romney Cave, which are maternity roosts located in Texas and New Mexico in the United States (Adams and Ammerman 2015; Bogan et al. 2017; England 2012). Movement patterns have been classified as commuting or foraging flights (Bogan et al. 2017). Commuting flights are characterized as long‐distance, high‐speed, and straight‐line movements occurring between roosts and foraging areas, and foraging flights are related to short‐distance, low‐speed, and meandering movements occurring in small patches of high resources, which have been described in other nectar‐feeding, frugivorous, and insectivorous bat species (Egert‐Berg et al. 2021; Goldshtein et al. 2020; Holland and Wikelski 2009; Hurme et al. 2019; Lavariega and Briones‐Salas 2016; Medellin et al. 2018; Roberts et al. 2012; Roeleke et al. 2020; Schloesing et al. 2020; Vleut et al. 2019; Voigt et al. 2017). Radiotelemetry and Passive Integrated Transponders (PIT tags) studies suggest that movement and activity patterns vary between age and sex groups. Larger foraging home ranges were observed for juveniles, presumably due to landscape exploration (England 2012), whereas adult females remained closer to the roost and commuted < 30 km to reach areas with high densities of floral resources in round trips ranging from 13.3 to 30 km each night (Bogan et al. 2017; England 2012). Specifically, lactating females spent shorter periods of activity outside the roost, which is associated with high energetic costs from nursing their young (Adams 2015). In contrast, studies using fluorescent powders, PIT tags, and GPS tracking conducted with their sympatric species, the Lesser long‐nosed bat ( *L. yerbabuenae* ), found that lactating females spent longer activity periods outside the roost and commuted longer distances, with 55.4 ± 17.2 km (mean ± SD) from their roost to foraging areas, reaching up to 200 km round trips per night (Goldshtein et al. 2020; Medellin et al. 2018; Rivera‐Villanueva et al. 2024). GPS tracking revealed fine‐scale movements in which bats typically return to the same foraging areas on consecutive nights and concentrate their foraging activity on a relatively small area of 0.14 ± 0.09 km^2^ with abundant floral resources (Goldshtein et al. 2020).

Mexican long‐nosed bats feed on a wide array of floral resources and are the main pollinators of agaves and columnar cacti across arid and semiarid habitats (Kunz et al. 2011; Sánchez and Medellín 2007; Valiente‐Banuet et al. 1996). Agaves are long‐lived succulent plants, native to North America with the highest species diversity in Mexico (Gentry 2003). Most agave species reproduce only once in their lifetime, and after investing substantial energy into producing a flowering stalk the plant dies (Gentry 2003; Trejo‐Salazar et al. 2016). The mutualistic relationship between agaves and nectar‐feeding bats is ecologically significant, as these plants not only provide essential nectar during critical periods such as lactation and migration, but also rely heavily on bat pollination for successful reproduction (Kunz et al. 2011; Moreno‐Valdez et al. 2004). Agave species remain a key component of Mexican long‐nosed bats diet, so the loss of these floral resources near the roosts is thought to have a detrimental effect on the population (Lear et al. 2024; Sánchez and Medellín 2007). Land use changes for agriculture and unsustainable harvesting of *Agave* spp. for the production of tequila, mezcal, and pulque (activities that involve harvesting agaves before they flower) are considered main threats contributing to the loss of food resources. However, the location and extent of the foraging areas remain unknown across most of the species distribution (Trejo‐Salazar et al. 2016; U.S. Fish and Wildlife Service 2018).

Understanding bats nightly movements is essential to properly assess their ecological interactions and conservation needs, especially in maternity roosts (Gómez‐Ruiz and Lacher 2017; Medellin et al. 2018; Medellín 2015; U.S. Fish and Wildlife Service 2018). The “Aguacatitla Tunnels” maternity roost is considered extremely important to ensure the conservation of the species, as it has maintained the highest number of births and remained stable since at least 2010 (Rojas‐Martínez et al. 2010; Zamora Vera 2015). Therefore, we tracked Mexican long‐nosed bats lactating females using GPS loggers and described their movement patterns and foraging areas locations. Nectar‐feeding bats adapt their foraging activity based on the spatio‐temporal distribution and availability of their food resources (Lear et al. 2024; Ober et al. 2005). In addition, nectar production and sugar concentration vary between species and along the night, so bats have to allocate considerable time to find the best sources of food or visit a large number of flowers, which may require covering large distances per night (Medellin et al. 2018; Moreno‐Valdez et al. 2004; Ober et al. 2005; Trejo‐Salazar et al. 2016). In this study, we aimed to address two key questions: How far can lactating females travel each night in search of food, and where are their foraging areas located? Given the great diversity of agave and cacti species within the region, and their ecological similarity with Lesser long‐nosed bats, we expected that Mexican long‐nosed bat lactating females would perform long‐distance flights, potentially reaching foraging areas located up to 50 km from the roost.

## 2 Materials and Methods

### 2.1 Study Region and Maternity Roost

**FIGURE 1:**
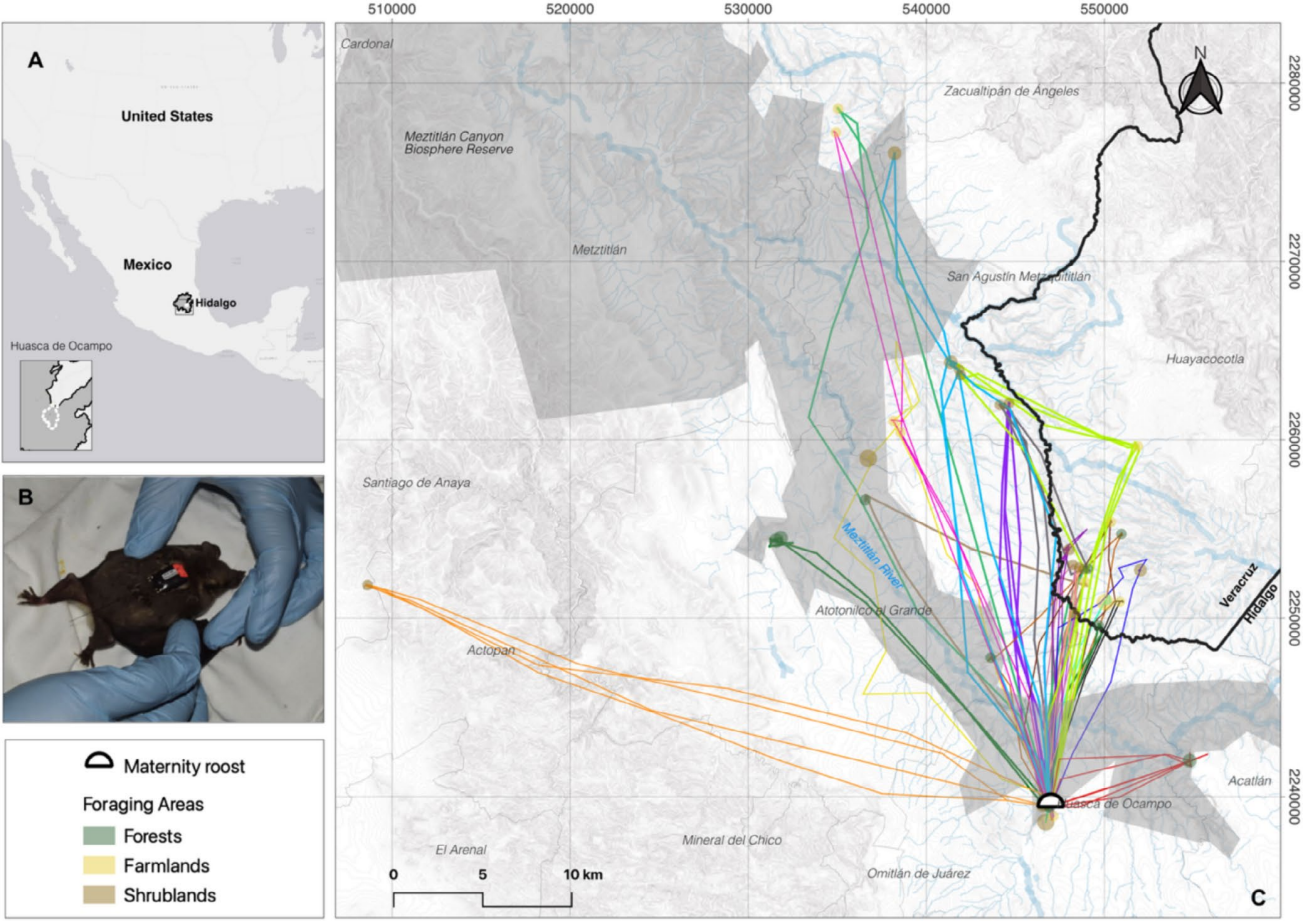
Movement tracks of 21 Mexican long‐nosed bats (lactating females) captured during springs of 2021 and 2022. (A) Location of Aguacatitla Tunnels maternity roost. (B) Mexican long‐nosed bat lactating female equipped with GPS logger. (C) Movement tracks and locations of the foraging areas of each tagged bat. Each individual movement track is represented by a different color, colored points represent foraging areas, whereas the bold black and gray lines represent state and municipal boundaries, respectively. The ESRI gray (light) base map from the QuickMapServices plugin was used.

“Aguacatitla Tunnels” maternity roost is located in the town of Aguacatitla, Huasca de Ocampo municipality, in the state of Hidalgo, Mexico (Figure 1A). This area is within the southern end of the Meztitlán Canyon Biosphere Reserve (MCBR), at an elevation of 2200 m, with rugged topography and a wide array of vegetation types that include pine‐oak and juniper forests at higher elevations, and tropical dry forests, shrublands, grasslands and riparian forests at the deepest part of the canyon system (CONANP 2003). Plants show Nearctic affinities with a high degree of endemism, and high densities of members of the families Asparagaceae and Cactaceae are widely distributed in the area (CONANP 2003; Rzedowski 2006).

Each year, the maternity colony is established in the darkest part of one of the main tunnels in an abandoned hydroelectric plant; the tunnel is approximately 100 m long, 6 m high, and 1.6 m wide, with an average temperature of 17.2°C and 100% humidity due to water circulating throughout the tunnel; and it is under the management of an ecotourism center by a cooperative association that embraces the conservation of bats, promoting controlled guided visits throughout the tunnels to observe the maternity roost as one of their main attractions (Rojas‐Martínez et al. 2010; U.S. Fish and Wildlife Service 2018; Zamora Vera 2015). This colony is relatively new, and it has been known since the early 2000s, with pregnant females arriving by mid to late March. The exact date the colony was formed is unknown, but according to the period of inactivity of the hydroelectric plant, it may have formed approximately 50 years ago (Rojas‐Martínez et al. 2010; U.S. Fish and Wildlife Service 2018). The colony size fluctuates as the season progresses, increasing until June when most of the females have given birth and newborns are still nonvolant (colony size estimates are up to 13,596 individuals). The colony size decreases in early August, when most individuals leave the roost and their migratory routes are unknown (Rojas‐Martínez et al. 2010; Zamora Vera 2015).

### 2.2 GPS‐Data Collection

During a total of five nights throughout May and June 2021 and 2022, we captured 29 Mexican long‐nosed bats, lactating females, and equipped them with small‐sized (1 g) Lotek Pinpoint 10 GPS store‐on‐board loggers (Lotek 2021). Bats were captured and handled following the Guidelines of the American Society of Mammalogists for the use of wild mammals in research and education (Sikes and The Animal Care and Use of the American Society of Mammalogists 2016). We used a six‐meter‐long mist net placed in parallel at the tunnel entrance; this way, we avoid blocking completely the exit of bats and reduce the number of captures, considering that during this period thousands of females leave the roost to forage each night. Besides, because there were no flying juveniles, we avoided separating them from their mothers. The net was opened at sunset and closed immediately after enough Mexican long‐nosed bats, lactating females weighing at least 30 g, were captured (GPS devices represented < 5% of the bats body mass). Individuals that did not meet these criteria were immediately released after capture. Captured bats were kept in cloth bags outside the tunnel for GPS placement; they were fed with sugary water and released within 30 min after being captured.

GPS loggers were adhered to the interscapular area with short‐term surgical glue so that they could be dropped in the next three nights. Due to their limited weight and battery, GPS loggers need to be retrieved to download data that include spatial information (Roeleke et al. 2020). In May 2019, we carried out pilot trials with dummy GPS loggers to evaluate attachment methods and success in recovering the real devices, achieving a 75% recovery success. For both pilot trials and tagging nights, we considered only lactating females because they must return to the maternity roost to feed their young, increasing the probability of dropping their devices inside the tunnel. The search for the GPS loggers was carried out at night, while the females left the roost for foraging. Since water on the tunnel floor can reach up to 10 cm in height, we used fluorescent powder (Medellin et al. 2018) on the bottom end of the loggers to ease the search using ultraviolet and white light lamps. Seven bats were tagged in 2021, and 22 were tagged in 2022, with an overall recovery success of 21 different movement tracks of Mexican long‐nosed bats lactating females.

We used two intervals for the GPS loggers to record bat locations. In 2021, locations were recorded every 15 min from 20:00 h to 05:30 h, whereas in 2022, the locations were recorded every 10 min from 20:30 h to 05:30 h. The schedule change for 2022 was considered in order to obtain movement data at finer scales; however, since foraging occurs in small areas, either of the two schedule rules worked in a similar way to record movement data, and we employed both years of movement data for the analysis. Spatial data from 21 GPS loggers were collected with at least one night movement track for each bat, five for 2021 and 16 for 2022. The downloaded files were processed using the PinPoint host application and DLC interface (Lotek Wireless, USA), which transformed the “swift” GPS fixes into regular WGS84 coordinates. Only 709 fixes out of 1343 were valid because the rest did not have sufficient satellites needed to establish a successful satellite connection. For the analysis, a four‐digit identification code was assigned to each movement track, using the first two digits to indicate the year of capture and the last two digits for the bat number (e.g., Bat 2101). The first activation and triangulation of GPS loggers was always done in the same location, so we excluded data points from 20:00 h to 21:30 h near the first fix of the night to avoid bias in data interpretation.

### 2.3 Path Segmentation and Movement Patterns

Preliminary data analyses were carried out to assess the structure and composition of movement data, the sampling regime, and data regularity (Edelhoff et al. 2016; Getz and Saltz 2008). We described movement patterns using First Passage Time (FPT) as the path segmentation method. FPT measures the search effort within an area at a given spatial scale, measuring the time it takes for an individual to enter and leave a circle of fixed radius *r* drawn around each location (Fauchald and Tveraa 2003). Because increased search effort is associated with foraging activity, this methodology is useful to identify foraging behaviors like Area‐Restricted Search (ARS), which has been documented in bat species that exploit patchy resources (Goldshtein et al. 2020; Hurme et al. 2019; McKenzie et al. 2009; Pinaud 2008; Schloesing et al. 2020; Voigt et al. 2017).

FPT values were calculated for each movement track using a radius of 300 to 500 m. We computed the relative variance of log‐transformed FPT values, defined as *S(r)* = *Var[log t(r)]*, where *t(r)* is the time lag between the first‐passage time forward and first‐passage time backward at radius *r* (Fauchald and Tveraa 2003). For each movement track, we identified the radius that maximized *S(r)*, as this peak indicates the spatial scale at which the animal increased its search effort. We selected the mean optimal radius 339 ± 62.99 m (mean ± SD, *n* = 21) across all individuals for the final FPT calculation. Two behavioral modes were classified using a threshold to the log‐transformed FPT values as follows: < 2.7141 values as commuting flight (CF) and > 2.7141 values as foraging flight (FF) (Fauchald and Tveraa 2003; Gurarie et al. 2016; Pinaud 2008; Rotger et al. 2020). Examples of path segmentation and *S(r)* are given in Figure S1. FPT calculations were performed using the functions “fpt” and “varlogfpt” from the “adehabitatLT” R package (Calenge 2006). Quantum GIS (QGIS‐LTR‐3.22 Bialowieza) was used for visual inspection and data manipulation of movement tracks.

We conducted a *post hoc* analysis to compare CF and FF segments using three additional metrics: step length, flight speed, and elevation. The step length is equivalent to the Euclidean distance between two consecutive relocations (Edelhoff et al. 2016; Gurarie et al. 2016); we used the sum of step lengths between relocations as total distance traveled per segment. Flight speed was estimated by dividing total distance traveled by time elapsed per segment. The average elevation for each segment was calculated using the Mexican Continuum of Elevations for the states of Hidalgo and Veracruz with 15‐m resolution in Quantum GIS (QGIS‐LTR‐3.22 Bialowieza). For the analysis, we used the mean values of each variable calculated per segment for each track. We then performed nonparametric Mann–Whitney *U*‐test to test for differences between CF and FF segments using PAST 4.15 (Hammer et al. 2001).

### 2.4 Trips and Foraging Areas Characteristics

Visual inspection of tracks revealed some bats made several trips during the night, which consisted mainly of commuting‐foraging‐commuting segments. Time lags of 30–60 min between the last location on its way to the roost and the following location leaving the roost allowed us to separate between trips, assuming that bats entered the roost between trips. For each round trip, we estimated total distance traveled and the mean flight speed. Foraging areas were defined from the FPT analysis, so each foraging segment was considered a foraging area. Foraging segments lasting at least 30 min (two or three consecutive relocations) were considered foraging areas, corresponding to an area where bats exhibit ARS behavior and actively search and consume floral resources (Fauchald and Tveraa 2003; Gurarie et al. 2016; Schloesing et al. 2020). Shorter foraging segments were not considered foraging areas for this analysis because brief visits in certain areas could be related to landscape exploration and location of potential foraging areas (Bogan et al. 2017; England 2012; Goldshtein et al. 2020).

The radius at which the relative variance of FPT peaked was used to estimate the size of each foraging area (Fauchald and Tveraa 2003; McKenzie et al. 2009; Pinaud 2008; Rotger et al. 2020). Additionaly, we calculated distance from the roost, search time effort (time spent by lactating females on foraging areas) and determined the habitat type for each foraging area. Habitat classification was based on a land cover map (INEGI 2021), by identifying the land cover type in which most foraging relocations occurred. Accordingly, habitat types were classified as follows: farmlands (for crops and grasslands areas), forests (for temperate and dry forests areas), or desert shrublands. Nonparametric Kruskal–Wallis tests were performed to test for differences across foraging area habitat types. These analyses were conducted using PAST 4.15 (Hammer et al. 2001) and Quantum GIS (QGIS‐LTR‐3.22 Bialowieza).

## 3 Results

We captured 29 Mexican long‐nosed bat lactating females and equipped them with GPS loggers to track their nightly movements. We recovered 21 devices with data from 21 different individual movement tracks covering an 8‐h period for one night (1.06 ± 0.36 nights, mean ± SD, *n* = 21) and a total of 589 filtered locations (28.05 ± 9.65 locations, mean ± SD, *n* = 21). Preliminary analyses indicated that all tracks were irregular, which means that there were certain time gaps in which the GPS loggers did not record the bat location (time gaps = 21.05 ± 13.68 min, mean ± SD, *n* = 21). These gaps appeared to be related to bats returning to the roost after visiting foraging areas, and 71% of the tracks presented time gaps during foraging activity (Figure S1).

FPT analysis revealed two movement patterns which were classified as commuting or foraging flights. Commuting flights had a total of larger step lengths (15.49 ± 8.99 km, mean ± SD, *n* = 21) compared to foraging flights (3.70 ± 2.40 km, mean ± SD, *n* = 21). Flight speed was higher in commuting flights (6.92 ± 1.74 m/s, mean ± SD, *n* = 21) than in foraging flights (1.49 ± 1.10 m/s, mean ± SD, *n* = 21), but the elevation was higher on foraging flights (1973 ± 145 m, mean ± SD, *n* = 21) than in commuting flights (1879 ± 117 m, mean ± SD, *n* = 21). These differences in step lengths, flight speed, and elevation were statistically significant (Mann–Whitney *U*‐test: step lenght, *U* = 10, *p* < 0.001; flight speed, *U* = 3, *p* < 0.001; elevation, *U* = 134, *p* = 0.031).

**FIGURE 2:**
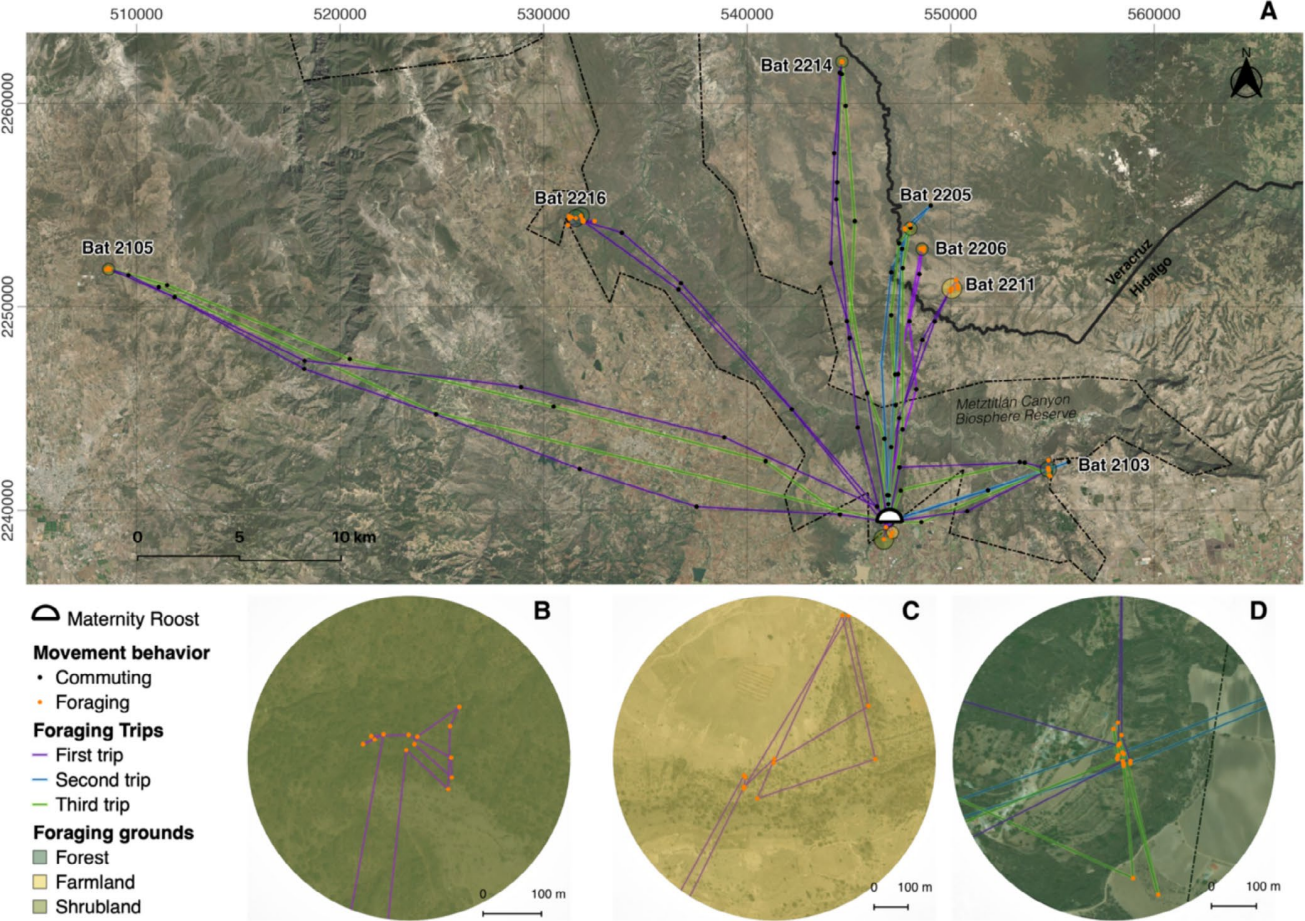
Directed foraging trips and relocations classified according to their movement behavior, cirular polygons represent the areas with the highest FPT values. (A) Movement tracks of bats visiting one main foraging area. (B) Shrubland foraging area for bat 2206, which displays foraging activity in a small spatial scale. (C) Foraging area for bat 2211 on a larger scale and farmlands surrounding the foraging relocations. (D) Foraging area for bat 2103, this bat visited the same area during three trips during one night. The ESRI satellite base map from the QuickMapServices plugin was used.

The total distance traveled per night varied among bats (Figure 1C), with a mean of 61.72 ± 37.22 km (mean ± SD, *n* = 21, range = 23.11 to 160.55 km), and a mean speed of 4.21 ± 0.98 m/s SD (range = 2.61 to 6.47 m/s). Bats performed between one and three round—trips throughout the night (Figure 2), with a mean of 1.67 ± 0.73 round trips (mean ± SD, *n* = 21). Most tracks consisted of a single complete round—trip, with a mean distance of 34.42 ± 28.56 km (mean ± SD, *n* = 10). Lactating females that performed a second complete round—trip typically covered longer distances (44.94 ± 23.12 km, mean ± SD, *n* = 8). In contrast, the shortest distances were associated with the third round—trip (26.29 ± 7.16 km, mean ± SD, *n* = 3). During these trips, bats visited up to four different foraging areas, especially those that made one or two complete round—trips. Detailed information on each track is presented in Table S1.

**FIGURE 3:**
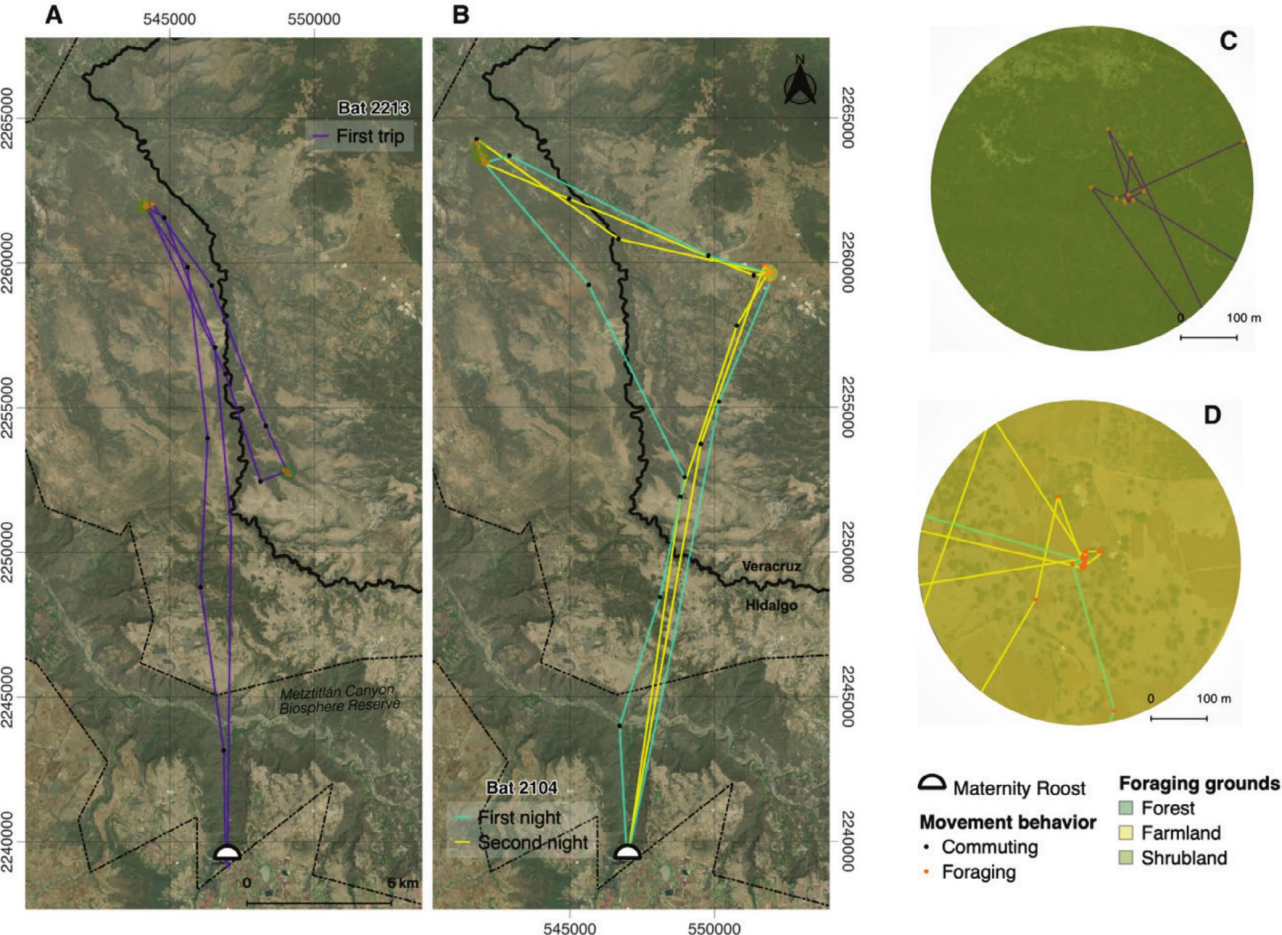
Stochastic foraging trips for bats visiting two foraging areas throughout the night. (A) Bat 2213 returning to the shrubland foraging area after visiting an alternative forest foraging area. (B) Bat 2104, the only movement track with complete data for two consecutive nights revisiting the same areas each night. (C) Shrubland foraging area for bat 2213. (D) Farmland foraging area for bat 2104; during the second night, this bat spent more time feeding in this area than in the shrubland area. The ESRI satellite base map from the QuickMapServices plugin was used.

Foraging activity during trips varied among bats; 62% of the bats performed stochastic movements and concentrated their activity within one foraging area and visited alternative areas during the rest of the night (Figure 3), whereas the rest performed directed movements and visited only one foraging area and revisited it throughout the night (Figure 2). Commuting from the roost to the foraging areas took 46% of the time bats spent moving during the night (2.30 h mean ± 1.01 h SD), whereas the remaining 54% of the time was spent in foraging activities (2.65 h mean ± 1.12 h SD), and 81% of this foraging time (2.16 h mean ± 1.14 h SD) was concentrated in one main foraging area. The highest peaks of foraging activity were concentrated from 22:00 to 01:00 h and from 02:00 to 04:00 h. Only four tracks exhibited time gaps of 30 min at foraging areas before bats commuted back to the roost or moved to another foraging area. Time gaps of 30 to 90 min were also observed between trips (Figure S1B,C).

We identified 37 foraging areas used by bats from Aguacatitla Tunnels maternity roost. With respect to habitat types, 46% of the foraging areas were located in shrublands, and the rest were located in forests and farmlands (27% each). Only 30% of the total foraging areas were located within the limits of the MCBR (forests = 70% and shrublands = 23%). The distance from the roost, area and search time varied between habitat types, but no significant differences were found among them (Figure S2). The mean distance from the roost to foraging areas was 16.41 ± 11.15 km (mean ± SD, *n* = 37), with farmlands and shrublands located from < 1 km to ~40 km from the roost and forests were located at closer distances < 1 km to 20 km (H(2) = 3.556, *p* = 0.169). As FPT relative variance represents a radius in which animals concentrate their search time effort, the mean area for foraging areas was 0.38 ± 0.16 km^2^, mean ± SD, ranging from 0.28 to 0.78 km^2^ (H(2) = 2.581, *p* = 0.206). However, 57% of the total foraging areas were small in size (0.28 km^2^), with bats feeding at smaller spatial scales, particularly in farmlands and shrublands. Finally, bats foraging in forests had the highest search time effort and spent a mean time of 1.38 ± 1.08 h (mean ± SD, *n* = 10), followed by those foraging in shrublands (1.30 ± 1.06 h, mean ± SD, *n* = 17) and farmlands (1.25 ± 1.01, mean ± SD, *n* = 10).

## 4 Discussion

In this study, we aimed to describe the nightly movement patterns and identify the foraging areas used by Mexican long‐nosed bat lactating females, an endangered nectar‐feeding species, at the only known maternity roost in central Mexico. Despite the limited sample size, GPS data obtained from 21 individuals movement tracks provided clear evidence that these bats can perform long‐distance flights throughout the night, reaching foraging areas up to 50 km from the roost. These results are consistent with our predictions and confirm that Mexican long‐nosed bats lactating females are capable of flying through extensive areas in search of floral resources during the energetically demanding lactation period. Moreover, our results support the idea that the region provides sufficient floral resources to sustain the maternity colony during spring and summer, as bats forage in native shrublands and cultivated areas.

### 4.1 GPS Tracking

Recording systems and sampling regime determine how well a trajectory reflects the actual movement path of an animal, which influences the spatial accuracy and frequency of relocations (Edelhoff et al. 2016). The use of GPS loggers provided higher‐resolution movement data compared to traditional VHF telemetry, allowing for fine‐scale tracking of individual bats. However, one of the main limitations of our study was the need to retrieve GPS loggers and their limited battery capacity, which recorded only one complete night for 20 bats and a single individual over two nights. Three key factors contributed to the successful recovery of most devices: (1) the selection of lactating females, which reliably return to the roost to feed their young; (2) the species cave‐roosting behavior, which increases site fidelity; and (3) the structural conditions of the maternity roost, which facilitated the detection and retrieval of dropped devices.

On the other hand, at the time of the study, no GPS devices with larger battery capacity met the weight requirements to be safely used on bats, which remains one of the technological barriers for tracking migratory bat species (Holland and Wikelski 2009). By adhering to recommended weight thresholds for tracking devices, we likely reduced additional stress in the individuals and minimized potential behavioral alterations. Notably, one individual in our study returned to the same foraging areas across two consecutive nights, suggesting behavioral consistency. The movement patterns we recorded were aligned with behaviors previously described for bats of the genus *Leptonycteris*, which exhibit no constraints for long‐distance commuting (Bogan et al. 2017; England 2012; Medellin et al. 2018; Ober et al. 2005). In particular, lactating females leave and return to the roost multiple times throughout the night (Fleming et al. 1998), and typically revisit the same foraging areas or shift to alternative ones depending on local floral resource depletion (Goldshtein et al. 2020; Ober et al. 2005).

### 4.2 Movement Patterns

Bat movements were classified into two main patterns, commuting and foraging, which were clearly distinguished by step length, flight speed, and elevation. Our results are consistent with previous descriptions of foraging behavior, characterized by meandering and repetitive movements on short timescales and at small spatial scales, with the animal changing course frequently and reducing its speed as it finds and moves between food items (Dingle and Drake 2007; Fauchald and Tveraa 2003). In contrast, commuting is an extended form of foraging in which longer to‐and‐from or round‐trip journeys are made regularly to spatially separated resource patches, roost sites, and other localities where specific activities occur (Dingle and Drake 2007). Our results show that bats commute across valleys to reach foraging areas, mainly located in mountainous regions. This behavior is consistent with movement patterns observed in other maternity roosts (Bogan et al. 2017; England 2012). Furthermore, species distribution models indicate that areas with higher abundance of *Agave* spp., key foraging resources for nectar‐feeding bats, are concentrated along mountain chains (Burke et al. 2019; Gómez‐Ruiz and Lacher 2017). Based on these findings, we confirmed that FPT analysis is a simple and effective method when first exploring animal movement, particularly useful when technical limitations exist such as restricted GPS battery life (Edelhoff et al. 2016; Gurarie et al. 2016; Hurme et al. 2019).

Many bats perform long commutes to foraging areas, with their movement patterns shaped by the predictability of food resources (Prat and Yovel 2020). Our results show that most Mexican long‐nosed bat lactating females made between one and three round‐trips per night, and that movements between trips were either stochastic or directed. Nectar‐feeding bats are able to remember the locations of multiple floral resources and have been suggested to have an extremely developed spatial memory (Voigt et al. 2017). When food location is predictable, bats tend to commute directly to their known foraging areas and then return directly to the roost. In contrast, when resources are ephemeral, bats search for food in a more stochastic way, covering much larger areas during foraging (Egert‐Berg et al. 2018). Most lactating females in our study visited alternative foraging areas during the night, which is similar to the behavior described for Lesser long‐nosed bats. Lactating females of this species commute to familiar foraging areas with high fidelity between consecutive nights and divide these areas into small foraging cores, gradually increasing the exploitation‐to‐exploration ratio of unvisited areas throughout the night (Goldshtein et al. 2020). In our study, females tended to perform longer commutes during their second trip; this suggests that they expanded their search to unexplored foraging areas.

Lactating females presented three foraging activity peaks during the eight‐hour period covered by our GPS loggers; however, as observed by Adams (2015), activity patterns varied among individuals. Our results align with roosting behavior described for Lesser long‐nosed bats, in which lactating females leave their young an average of three times each night (Fleming et al. 1998), and some extend their foraging bouts while other females stay in the roost to care for the young (Rivera‐Villanueva et al. 2024). Nectar‐feeding bats visit several flowers and consume nectar until they satiate, then they rest in order to digest and then visit flowers again; in this way, they can visit up to 1000 flowers per night (Tschapka and Dressler 2002). Some tracks had temporal gaps during foraging activity, suggesting the possible use of temporal night roosts such as caves or man‐made structures with little disturbance near foraging areas, as observed by Adams (2015), Bogan et al. (2017) and England (2012). In our study, GPS signal loss might be related to the terrain's topography; however, although we were not able to visit all identified foraging areas, we did observe nectar‐feeding bat activity inside abandoned or unfinished houses near some foraging areas. Additionally, the longest trip made by Bat 2105 coincided with the location of Xoxafi Cave, which is known for being used by both Mexican long‐nosed bats and Lesser long‐nosed bats during the summer (U.S. Fish and Wildlife Service 2018).

### 4.3 Foraging Areas

Lactating females spent > 50% of their time outside the maternity roost foraging in small areas composed mainly of shrublands, followed by farmlands and forests. The foraging areas were concentrated northwards from the maternity roost, mainly in the southwestern region of Huayacocotla municipality, in the state of Veracruz, along with San Agustín Metzquititlán and Zacualtipán municipalities, in the state of Hidalgo at 13, 25, and 40 km from the roost respectively. These areas are well known for having large extensions of columnar cacti and both wild and cultivated *Agave* spp. growing along the complex topography of the region (CONANP 2003; Eguiarte et al. 2008; Torres‐García et al. 2019; Trejo‐Salazar et al. 2015). As we predicted, lactating females can find profitable nectar resources within at least 50 km of the maternity roost; however, as observed by England (2012) in the Emory Cave maternity roost, most females chose to forage in areas closer to the roost (< 20 km), although larger commutes (> 20 km) had also been described in Romney Cave postmaternity roost by Bogan et al. (2017). Commuting to more distant foraging areas even when closer alternatives are available is likely related to the high density of individuals within the roost, which may increase competition for floral resources and is reduced by spatial spreading (Egert‐Berg et al. 2018; Goldshtein et al. 2020; Horner et al. 1998).

Fleming et al. (1998) studied the behavior of Lesser long‐nosed bats in a maternity colony at Organ Pipe Cactus National Monument, Arizona, USA. They found that the colony comprises bats from different regions of the species distribution, arriving at different times and from various roosts. This suggests that bats reach the roost by following nectar corridors that connect them to the maternity colony. Under these conditions, each group of bats appears to be familiar with the route it used to arrive and with the floral resources available along it. Initially, bats likely travel these routes each night to forage and to search for new nearby foraging areas. On the other hand, evidence suggests that Mexican long‐nosed bats low genetic structure may result from the long‐distance movements of most females, and that female migratory movements may not be entirely philopatric, with many individuals potentially switching roosts upon return (Trejo‐Salazar et al. 2025). In our study, the different distances and directions traveled per bat may reflect the regional heterogeneity of the colony's composition and could explain the variation in distances traveled to foraging areas.

There were no significant differences observed among the three habitat types with respect to distance from the roost, spatial scale, or time spent in the foraging areas. This might be because, given that flight is energetically costly, and that, in general, bat movements are dependent on the spatial distribution, richness, and availability of their food resources, they are capable of flying long distances to reach profitable foraging areas (Burke et al. 2019; Rainho and Palmeirim 2011; Voigt et al. 2017). Considering the above, there are at least 14 species of bat‐pollinated agave and cacti growing along the MCBR, which bloom in May, but nectar production is variable in each species, and lactating females must drink ~18 mL of nectar per night to satisfy their energetic demands (Ayala‐Berdon et al. 2013; Goldshtein et al. 2020; Trejo‐Salazar et al. 2016). Studies on bat visitation rates over floral resources have described a positive relationship with the number of available open flowers (Goldshtein et al. 2020; Lear et al. 2024) and highlight that bats apparently choose to forage on clusters of plants with an early blooming stage, presumably because they may contain more nectar or nectar with higher sugar concentrations (Lear et al. 2024). Overall, shrublands were the preferred habitat among bats, possibly due to a higher diversity and quality of agave and cacti resources. Although we were not able to assess the diversity or availability of food resources used by lactating females, we conducted field visits in the most frequently visited areas and confirmed that foraging areas were located in inaccessible foothills with considerable clusters of blooming agaves. Moreover, the surrounding areas were covered by large amounts of cultivated agaves mainly used for pulque production, fodder for livestock, and household consumption.

Previous studies in Aguacatitla Tunnels indicate that common items in Mexican long‐nosed bats diet include several wild agaves and columnar cacti, with *Agave salmiana* as the most important component of its diet (Martínez‐Bautista 2019). This is the most commonly used agave species in agroforestry systems in central Mexico, including homegardens, live fences, or terraces (Torres‐García et al. 2019); its inflorescence has up to 150 flowers that produce high amounts of nectar all day, so it functions as patches of resources for nectar‐feeding organisms (Gómez‐Aíza and Zuria 2010; del Martínez Rio and Eguiarte 1987). Additionally, its flowering period coincides with the presence of the maternity colony in central Mexico, which might explain the use of farmlands and forests as foraging areas observed in this study. In forests, bats spent more time foraging likely because they needed to visit more flowers to meet their energetic demands. Whereas in farmlands, which were the most distant areas from the roost, they forage at the smallest spatial scales, likely because of the aggregated nature of the floral resources. Bat 2104 movement track was the only track for which we had data for two consecutive nights, with the lactating female using the same farmland and shrubland foraging areas. This finding is consistent with previous studies on the foraging behavior of Lesser long‐nosed bats, which tend to revisit the same foraging areas during consecutive nights (Goldshtein et al. 2020), and alternating foraging areas has been associated with the end of nectar production by local flowers (Ober et al. 2005). Although more rigorous landscape analysis is needed, the above findings suggest that farmlands and forests areas may function as “stepping stones” food resources to reach particularly rewarding foraging areas.

## 5 Conclusions

The use of simple GPS devices allowed us to describe fine‐scale foraging movement patterns for lactating females of the endangered Mexican long‐nosed bat in central Mexico. Our results demonstrate that these animals are capable of flying long distances each night to find adequate nectar resources, using several foraging areas located in the foothills of shrublands, farmlands, and forests habitats. During foraging trips, they move through extensive areas, connecting agave and cacti populations that other pollinators with much smaller ranges of movement cannot reach. Considering that one main conservation effort in the species recovery plan includes the protection of foraging areas, our results demonstrate the importance of integrating movement ecology in monitoring for the Mexican long‐nosed bat and other bat species, given that most bat foraging activity was concentrated outside the protection of the Metztitlán Canyon Biosphere Reserve. As human environments, such as agroforestry systems, are used by lactating females, future studies aiming to explore the relationships among nectar‐feeding bats, cultivated agaves, and agave handlers may improve our understanding of the importance of these habitats for the species in central Mexico. These studies will also aid in the design of reforestation plans for agave and cacti pollinated by bats to increase foraging resources and maintain migratory routes for the species.

## Author Contributions

**Paulina Soriano‐Varela:** conceptualization (equal), data curation (equal), formal analysis (equal), methodology (equal), writing – original draft (equal), writing – review and editing (equal). **Ana Ibarra‐Macías:** conceptualization (equal), funding acquisition (equal), methodology (equal), writing – review and editing (equal). **Alberto E. Rojas‐Martínez:** conceptualization (equal), methodology (equal), writing – review and editing (equal). **Claudia Elizabeth Moreno:** methodology (equal), writing – review and editing (equal). **Iriana Zuria:** methodology (equal), writing – review and editing (equal).

## Disclosure

*Statement on inclusion*: our study brings together a group of authors composed mostly of women, who are at different stages of their career. The group includes a graduate student, two research professors from a state public university, a retired research professor, and a regional representative of an international non‐governmental organization. All authors were engaged early on with the research and study design to ensure that the diverse sets of perspectives they represent were considered from the beginning. Mexican long‐nosed bats are distributed between Mexico and the United States, so whenever relevant, literature published by scientists from the *Nivalis Conservation Network* was cited; efforts were made to consider relevant work published in the local language. During the fieldwork, we worked together with local residents who manage the ecotourism development in which the maternity roost is located, sharing the research results and promoting bat conservation.

## Conflicts of Interest

The authors declare no conflicts of interest.

## Supporting information


**Data S1:** ece372055‐sup‐0001‐Supinfo01.docx.

## Data Availability

The datasets generated and/or analyzed during the current study are available in the Movebank Data Repository, https://doi.org/10.5441/001/1.696 (Soriano‐Varela et al. 2025).
