# Supplementary material for: Foraging Movement Patterns of Lactating Mexican Long‐Nosed Bats in Central Mexico
Source: Ecol Evol. 2025 Sep 2;15(9):e72055. doi: 10.1002/ece3.72055 (PMC12404700; doi:10.1002/ece3.72055)
Supplement: Supplementary file 1 — Data S1: ece372055‐sup‐0001‐Supinfo01.docx. [file ECE3-15-e72055-s001.docx]

Supplementary material – Figures and Tables

**Foraging movement patterns of lactating Mexican long-nosed bats in central Mexico**

Paulina Soriano-Varela, Ana Ibarra-Macías, Alberto E. Rojas-Martínez, Claudia Elizabeth Moreno and Iriana Zuria


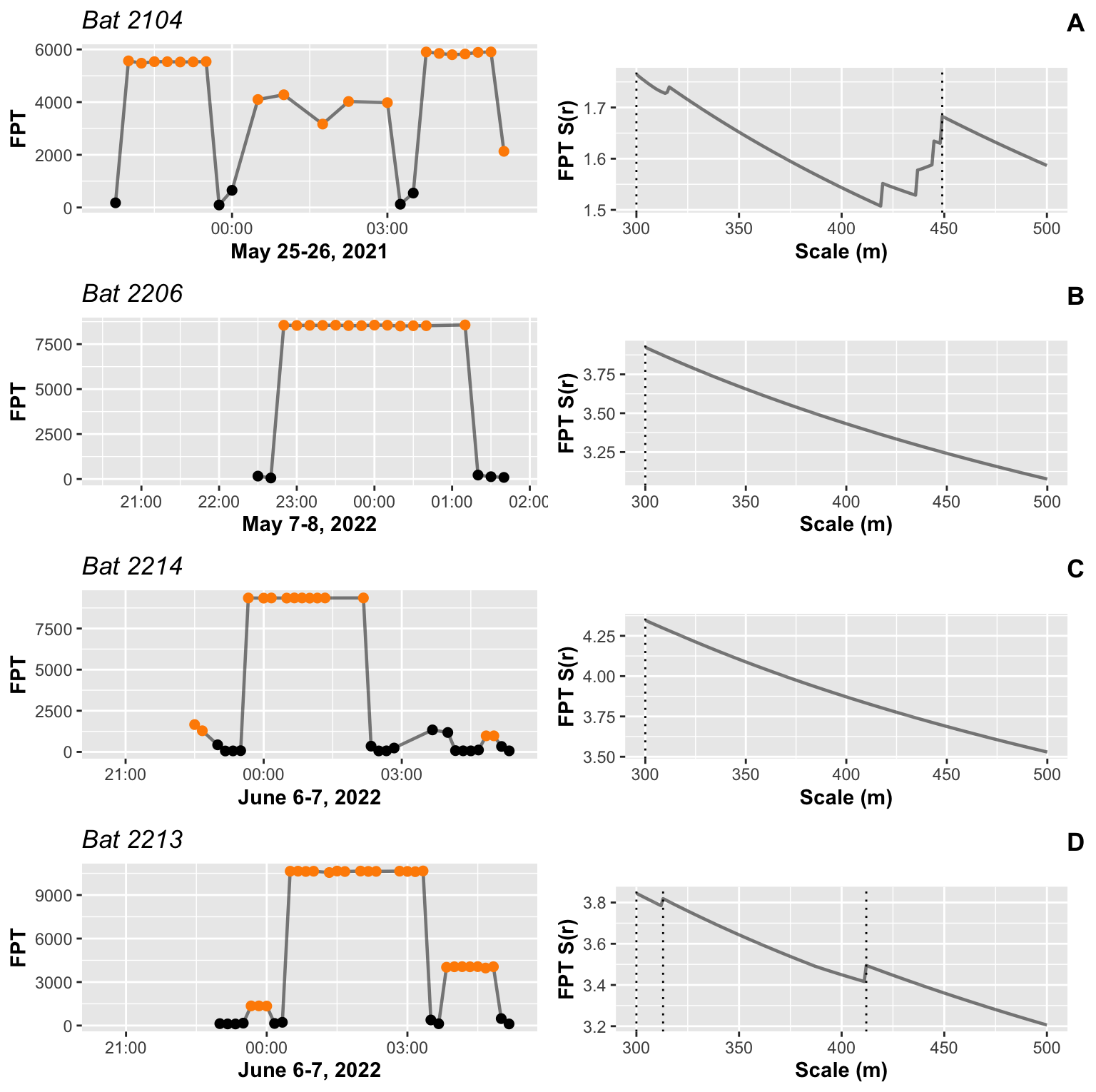


**Figure S1.** Examples of FPT analysis for different Mexican long-nosed bats lactating females. On the left, FPT is the time in seconds it takes for an individual to enter and leave a virtual circle of fixed radius drawn around each location. High FPT values are associated with area-restricted search behavior and considered as foraging segments (orange dots); whereas lower and variable FPT values as commuting segments (black dots). Gaps between locations represent the absence of record from the following location, which occured after foraging activity (B), and between trips with bats returning to the roost (C). On the right, peaks in relative variance of FPT S(r) denote the radius or spatial scale of foraging activity (dashed lines). Some bats displayed several ARS behavior at different spatial scales (A and D), but the highest value remains at a radius of 300 m.


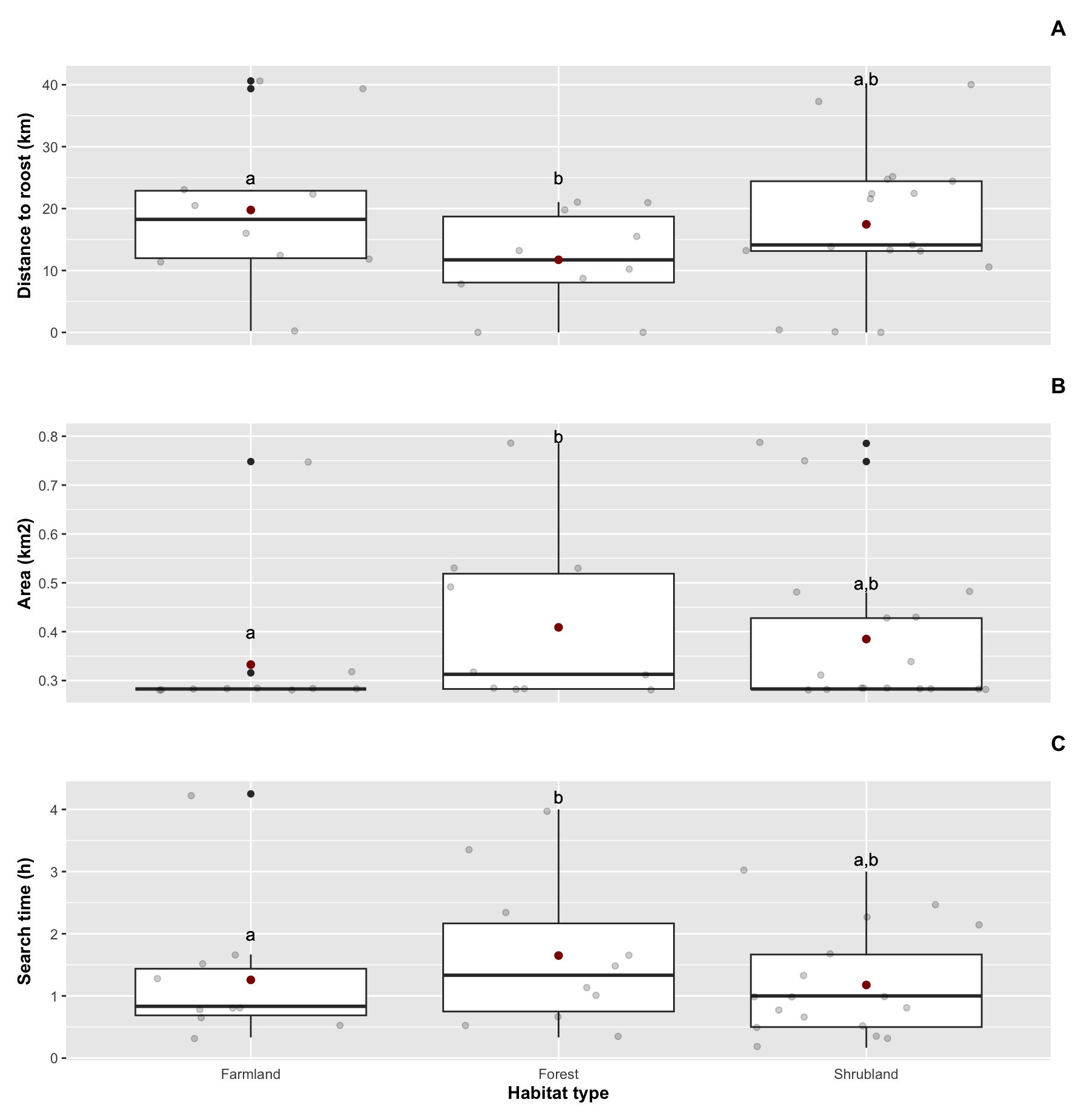


**Figure S2.** Foraging areas characteristics between habitat types: A) distance to the roost (H(2)=3.556, *p*=0.169), B) estimated area for foraging areas (H(2)=2.581, *p*=0.206), and C) search time effort spent on foraging areas (H(2)=1.059, *p*=0.587). The mean values are displayed by the dark red circles.

**Table S1.** Characteristics of the 21 Mexican long-nosed bats lactanting females movement tracks. All data corresponds to one night of tracking except for Bat 2104, which corresponds to the average values from two nights of tracking. ID = Classification code for each one the bats; Relocs = number of relocations per track (filtered), dt= average time interval between locations (minutes). Distance travelled per trip and total distance travelled per night is given in kilometers. Mean speed = average speed of total distance travelled, given in m/s. FPT S(r) = Relative variance in FPT, average radius at which all ARS were best identified; FA= total foraging areas visited per bat during the night.

| **ID** | **Relocs** | **dt** | **Trip 1** | **Trip 2** | **Trip 3** | **Total distance** | **Mean speed** | **FPT S(r)** | **FA** |
| --- | --- | --- | --- | --- | --- | --- | --- | --- | --- |
| **2101** | 26 | 21.60 | 80.38 | - | - | 80.38 | 4.77 | 300 | 4 |
| **2102** | 29 | 37.04 | 78.84 | 53.18 | - | 132.02 | 3.90 | 391 | 2 |
| **2103** | 28 | 20.55 | 19.36 | 18.73 | 18.66 | 56.74 | 2.61 | 395 | 1 |
| **2104** | 48 | 61.56 | 63.34 | - | - | 63.34 | 3.09 | 300 | 3 |
| **2105** | 23 | 25.23 | 81.61 | 78.95 | - | 160.55 | 6.47 | 300 | 1 |
| **2201** | 19 | 28.33 | 3.09 | 21.81 | - | 24.91 | 3.23 | 300 | 1 |
| **2202** | 18 | 27.65 | 0.30 | 34.83 | - | 35.12 | 3.84 | 300 | 1 |
| **2203** | 36 | 14.00 | 2.98 | 65.28 | - | 68.26 | 2.63 | 500 | 2 |
| **2204** | 22 | 51.61 | 1.38 | 32.10 | - | 33.48 | 3.51 | 369 | 2 |
| **2205** | 23 | 20.91 | 0.32 | 29.92 | 32.85 | 63.10 | 4.31 | 329 | 1 |
| **2206** | 25 | 13.33 | 28.41 | - | - | 28.41 | 4.14 | 300 | 1 |
| **2207** | 23 | 57.41 | 0.24 | 27.65 | 27.35 | 55.23 | 5.64 | 300 | 1 |
| **2208** | 30 | 16.55 | 27.79 | 86.09 | - | 113.87 | 4.08 | 300 | 2 |
| **2209** | 34 | 16.36 | 36.67 | - | - | 36.67 | 3.22 | 300 | 2 |
| **2210** | 14 | 25.38 | 23.11 | - | - | 23.11 | 3.99 | 314 | 2 |
| **2211** | 20 | 28.42 | 29.41 | - | - | 29.41 | 5.35 | 488 | 2 |
| **2212** | 13 | 29.16 | 25.36 | - | - | 25.36 | 4.74 | 300 | 1 |
| **2213** | 42 | 12.93 | 70.99 | - | - | 70.99 | 4.41 | 300 | 2 |
| **2214** | 36 | 45.68 | 47.86 | 45.79 | - | 93.64 | 4.69 | 300 | 2 |
| **2215** | 37 | 13.06 | 48.47 | - | - | 48.47 | 4.76 | 317 | 2 |
| **2216** | 43 | 11.90 | 52.94 | - | - | 52.94 | 4.97 | 410 | 2 |
